# Pretreatment with melatonin improves ovarian tissue cryopreservation for transplantation

**DOI:** 10.1186/s12958-021-00705-4

**Published:** 2021-02-03

**Authors:** Marcos Eiji Shiroma, Luciana Lamarão Damous, Fernanda Pereira Cotrim, Cristiane Lima Roa, José Cipolla-Neto, Russel Joseph Reiter, Edmund Chada Baracat, José Maria Soares

**Affiliations:** 1 Faculdade de Medicina FMUSP, Universidade de Sao Paulo, Av. Dr. Arnaldo, 455 - Cerqueira César, São Paulo, SP CEP 01246-903 Brazil; 2 Instituto de Ciencias Biomedicas ICB, Universidade de Sao Paulo, Av. Prof. Lineu Prestes, 1374 - Butantã, São Paulo, SP CEP 05508-000 Brazil; 3 University of Texas, Health Sciences Center, 7703 Floyd Curl Dr, San Antonio, TX 78229 USA

**Keywords:** Melatonin, Ovary, Transplantation, Fertility preservation, Cryopreservation, Tissue preservation

## Abstract

**Backgroud:**

Melatonin has anti-inflammatory and antioxidative actions at the mitochondrial level. This indole-containing molecule may protect ovarian grafts during the process of cryopreservation. Therefore, we aimed to determine whether melatonin pretreatment improves rat ovarian graft quality.

**Methods:**

Twenty-six female rats were allocated to two study groups of thirteen animals each: 1) control group: ovaries cryopreserved using the standard protocol; and 2) melatonin group: ovaries cryopreserved in a medium with melatonin. Ten rats of each group were submitted to 24-h freezing, and whole ovaries autologous and avascular transplantation with retroperitoneal placement. After postoperative (PO) day 15, daily vaginal smears were obtained for estrous cycle characterization. Between PO days 30 and 35, the animals were euthanized and ovarian grafts were recovered for histological and immunohistochemical (Ki-67, cleaved caspase-3, TUNEL, von Willebrand factor, estrogen, and progesterone receptors) analyses. The ovaries of the three remaining rats from each group were studied immediately after thawing to assess the effects of cryopreservation. ANOVA and Tukey’s tests were used and the rejection level of the null hypothesis was set at 0.05 or 5% (*p* < 0.05).

**Results:**

Melatonin promoted faster restart of the estrous cycle and increased the expression of mature follicles, collagen type I, von Willebrand factor, Ki-67, and cleaved caspase-3 on corpora lutea and estrogen receptors in the ovaries as compared to control. There was a reduction in apoptosis by TUNEL on follicles, corpora lutea, and collagen type III.

**Conclusion:**

Based on the evaluated parameters, melatonin may promote the quality of ovarian grafts. Reproductive function enhancement should be further studied.

## Backgroud

Recent advances in the treatment of oncological diseases have resulted in significantly improved survival of young patients with cancer [1–3], with a cure rate of over 90% [4]. In the USA, yearly estimates run as high as 60,000 new cases of cancer in women younger than 40 years of age; of these, 4000 are prepubertal children and adolescents [5]. However, both chemotherapy and radiotherapy may impair the reproductive future of these patients [1, 3–7]. Premature ovarian failure is estimated to occur in up to 68% of the women treated with alkylating agents for breast cancer during menacme, 38–57% of those aggressively treated with cytotoxic chemotherapy and radiotherapy for lymphomas, and in over 90% of patients undergoing a conditioning regimen for bone marrow transplantation [1–4]. Radiotherapy is also known to destroy the follicle reserve [1, 4, 5]. Fertility preservation is a major cause of concern in young women.

Preservation of the fertility of women is a challenge; likewise, the offer of the best chances of maternity to patients at risk of premature, induced, or iatrogenic menopause [3, 4]. Such an offer may be made not only to patients with malignant diseases, but also to those with sickle cell anemia and thalassemia, those requiring a bone marrow transplant, patients with lupus, or those with lesions leading to bilateral oophorectomy, such as recurring cysts [1–4], all of which entail reproductive failure. These are further reasons for improving the technique.

The options for maintaining reproductive capacity include oocyte, embryo, and ovarian tissue freezing [3–5]. Ovarian tissue cryopreservation is the only technique still deemed experimental. It allows the storage of a large quantity of primordial and primary follicles quickly and at any phase of the menstrual cycle [1, 6]. It is the only option for preserving fertility in prepubertal girls [1, 2, 4, 5, 7]. In cryopreserved ovarian tissue transplantation, one of the major difficulties to surmount is the improvement in the vascular bed of the receptor area [4–6], given the potential occurrence of ischemic lesions over the time it takes for graft revascularization [1, 2, 6, 7]. This lesion results in fibrosis and apoptosis, which affect the follicle survival rate and graft lifespan, both of which are key in fertility restoration [1, 7]. In addition, follicle activation in the graft accelerates after transplantation, leading to discrepancies in cell maturation in the granulosa and oocyte development, possibly due to hypoxic stress and lack of anti-Mullerian hormone in the graft [4]. Lastly, the freezing-thawing process induces structural and morphological changes, especially in the theca cell layer [4]. The main reasons for the limited quality of the ovarian graft are inflammatory processes and oxidative stress, which damage cells and mitochondria. These issues are the target of melatonin action [8].

Treatments such as ischemic preconditioning [9] and cell therapy with stem cells [10] have been studied by our group, with moderate benefits. Therefore, additional investigations are needed to improve ovarian transplantation. One hypothesis for the impaired outcome is the presence of factors capable of interfering with tissue response; the chief among them is a large number of free radicals derived from the procedure [1]. In this experimental research scenario, new techniques have been proposed, the most prominent of which is melatonin administration for reducing oxidative stress. Melatonin acts as a free radical scavenger with vast antioxidant and anti-apoptotic functions [6, 11–13]. Melatonin action diminishes free radical-induced lesions in several diseases, including cancer, and also inhibits the mitochondrial apoptotic pathway as it reduces BCL2 expression and cleaved caspase-3 activity [6, 14]. The antioxidant action of melatonin is more potent than that of vitamin C or E, as even its metabolites act as free radical scavengers, in a process called the cascade effect [12, 13]. In addition to acting directly against free radicals, melatonin activates antioxidant enzymes, such as superoxide dismutase, glutathione peroxidase, and catalase [12–14]. The surplus free radicals interact with lipids, proteins, and nucleic acids, causing the loss of membrane integrity, functional and structural changes in proteins, and nucleic acid lesions [13, 15].

Melatonin action is also recognized in diverse biological functions, such as the circadian cycle control and anticancer action and those related to the reproductive system (ovarian activity, pregnancy, and delivery) [11, 13] and neuroendocrinology, cardiology, and neuroimmunology [15]. It is a hydrophilic and lipophilic molecule [11, 13, 15] and easily diffuses in various subcellular compartments such as membranes, cytoplasm, nucleus, and mitochondria [11–13]. There is evidence of functional enhancement in human thawed sperm quality when melatonin is applied to the cryopreservation medium [16]. Therefore, melatonin may favor cell survival. However, it is not clear whether this effect may occur during the cryopreservation of tissue fragments such as ovarian grafts. Thus, this study aimed to evaluate rat ovarian grafts with melatonin added to the culture medium prior to cryopreservation.

## Materials and methods

The study was approved by the local Ethics Committee on the Use of Animals (CEUA-FMUSP 024/15). Sample size calculation was determined using Altman Normogram [17], and data from the results of a previous publication regarding melatonin treatment and follicular expression of vitrified ovarian grafts [18] comparing control and study groups. Sample size result was 20 animals for 2 groups considering 80% power and statistical significance of 5%. Therefore the research animal group consisted of 26 adult female Wistar rats (*Rattus norvegicus albinus*) aged 3 months and weighing approximately 250 g each. The animals were kept under proper conditions of temperature and feeding as well as under a controlled light/dark cycle of 12/12 h. Only animals with three regular estrous cycles were included.

The animals were allocated to two study groups (*n* = 13 each), namely, control and melatonin. In the control group, slow cryopreservation was performed according to the standard protocol using the M2 culture medium, dimethyl sulfoxide (DMSO) [19], and ethyl alcohol vehicle, whereas in the melatonin group, melatonin (Sigma Aldrich, Saint Louis, MO, USA) was added to the medium at a concentration of 10^− 7^ M [12]. Three animals from each group underwent graft analysis immediately after thawing from cryopreservation to verify the potential differences between groups induced up to the freezing-thawing process itself.

In both groups, the ovaries underwent slow cryopreservation and were kept in liquid nitrogen (N_2_) for 24 h. Thawing took place at room temperature (25 °C). The ovaries were implanted in the retroperitoneum of their respective donors, one on each side of the aorta, without anastomosis, and fixed using a simple stitch with an unabsorbable thread (nylon 4–0).

### Estrous cycle control

At the beginning of the experiment, vaginal smears were obtained daily, always at the same time (8–10 a.m.), to characterize the estrous cycle using the Shorr-Harris technique [20, 21]. Only animals with regular estrous cycles of 4–5 days were used; diestrus was the standard phase for the surgical procedures (oophorectomy for cryopreservation and euthanasia).

Daily (8–10 a.m.) vaginal smear collection was resumed from postoperative (PO) days 15–30, and euthanasia was performed as the animals entered the diestrus phase (PO day 30–35).

### Protocol for anesthesia

After being weighed, the animals were anesthetized with xylazine (15 mg/kg) and ketamine (60 mg/kg) administered via intraperitoneal injection [22].

### Oophorectomy protocol

After a median longitudinal opening of the abdominopelvic cavity, the ovaries were removed bilaterally and washed with saline (0.9% NaCl). The procedure was performed between 9 and 10 a.m.

### Cryopreservation and thawing protocol

The ovaries were placed in 1.2-mL cryotubes with 1 mL of 1.4 M DMSO as a cryoprotectant and the M2 medium with or without melatonin added to the medium, depending on the study group, and kept at room temperature for 5 min. Slow-freezing was performed using the CL-8800 temperature controller and the Cryogenesis software, and then controlling the freezer from 25 to 10 °C at 1 °C/min and to -7 °C at 0.5 °C/min, keeping the temperature for 5 min. Next, the temperature was lowered to -55 °C at 0.5 °C/min. At this point, the ovaries were transferred into liquid N_2_ at -196 °C and kept for 24 h [19].

The cryotubes were thawed at room temperature until all the ice melted (15–20 min). The tissue was then transferred to 5 mL of TL-HEPES at room temperature for 10 min, while it was gently agitated to promote DMSO efflux. The tissue was kept in TL-HEPES at 37 °C until transplantation [19].

### Collection and analysis of the material

The ovarian grafts were recovered and cut in half for analysis. The rats were euthanized with a lethal dose of the previously employed anesthetics. Imaging and measurements were performed using a computer system comprising a light microscope (Carl Zeiss) adapted to a high-resolution camera (Axio Cam MRC, Carl Zeiss) and a color video monitor. The measurements were taken with an image analysis software program (AxionVision REL 4.6, Carl Zeiss). Counting was always performed using four fields per animal.

The analyses comprised the following:

1. Estrous cycle

With the animal immobilized, the vaginal smear was first obtained using a swab impregnated with saline solution and then placed on a standard slide for subsequent staining using the Shorr-Harris technique [23]. The slides were subsequently analyzed using a light microscope at 10× and 40× magnification. The phases of the estrous cycle were determined according to the proportion of cells observed in the smears as follows: 1) proestrus, the predominance of nucleated epithelial cells, 2) estrus, the predominance of non-nucleated keratinized cells, and 3) diestrus, an equal proportion of leukocytes and nucleated keratinized epithelial cells [23].

2) Histology

To assess follicle development, the ovarian follicles were counted and then categorized into developing follicles, regardless of their stage, and atretic follicles. The former was classified according to the degree of maturation as follows: immature follicles (including primordial, primary, and secondary), mature follicles (with a single voluminous antrum), and corpora lutea. For counting purposes, the ovarian follicles comprised both viable and atretic follicles as well as both normal and degenerating corpora lutea [24].

Blood vessel count was performed in a 100-square-micrometer area in five randomly selected fields and analyzed by two independent observers.

For evaluation of fibrosis, the slides were stained with picrosirius red, and measurements were taken in eight fields per animal in the ovarian stroma, with a magnification of 400×. The results are expressed as a percentage of the positive area (unit/mm^2^).

The evaluation of the slides was conducted at our Medical Investigation Laboratory (LIM-58). For quantification of the parameters evaluated, the images were captured using a high-resolution camera (AxioCam-MCR, Carl Zeiss) adapted to a light microscope (Axiolab, Carl Zeiss) adjusted to the 40× objective lens [19].

3) Immunohistochemistry

Slides with sections of the ovarian grafts were stained using immunohistochemistry to measure the von Willebrand factor (AB6994, 1:100, Abcam Inc., Cambridge, MA, USA), Ki67 (M724001–2, 1:100, Dako North America Inc., Carpinteria, CA, USA), cleaved caspase-3 (SANT-SC-1226, 1:100, Santa Cruz Biotechnology, Santa Cruz, CA, USA), and TUNEL (terminal deoxynucleotidyl transferase (TdT)-mediated dUTP nick-end labeling, Roche, Indianapolis, IN, USA). Hormonal receptor expression for estrogen (E1644, 1:50, Springe Bioscience Corporation, Pleasanton, CA, USA) and that for progesterone (AB51896, 1:30, Abcam Inc., Cambridge, MA, USA) were also studied. All samples were prepared according to the manufacturer’s instructions.

The microscopy images were obtained using a computer program (Leica DM2500) and quantifications were performed using the LeicaQWin V3 program. The red-brown coloration of the cytoplasm and nucleus of granulosa cells and antral follicles (for apoptosis and Ki-67) or stroma (for fibrosis and expression of endothelial cells) was considered as positive expression and any other color, as negative. Hormone receptors were evaluated in both the stroma and follicular cells. The analysis was performed in eight different fields per animal at 400× magnification, and the results are expressed as a percentage of the positive area (unit/mm^2^). The interpretation was performed by two independent and blinded investigators.

### Statistical analysis

The data from each group of animals were analyzed according to the type of variables. The ANOVA and Tukey’s tests were used, and the rejection level of the null hypothesis was set at 0.05 or 5% (*p* < 0.05).

## Results

### Evaluation after freezing-thawing

Three animals from each group were analyzed immediately after the freezing-thawing process to verify the effects of melatonin specifically on the cryopreservation process. Histological assessment of immature follicles, mature follicles, corpora lutea, and blood vessels showed no significant difference between the control and melatonin groups (Table 1).

**Table 1:** Histological analysis

	Control	Melatonin	*P*-value
Immature Follicles	10.33 ± 2.90	4.66 ± 0.88	0.13
Mature Follicles	11.00 ± 3.51	5.33 ± 1.20	0.20
Corpora Lutea	10.00 ± 3.46	10.33 ± 4.70	0.95
Blood vessels	4.66 ± 1.45	2.66 ± 1.76	0.43

Histological analysis comparing the use of melatonin added to the cryopreservation medium or not, in rat ovarian slide, freezing-thawing group (*n* = 3 in each group).

Immunohistochemical assessment of the freezing-thawing group showed no significant difference in cleaved caspase-3 and TUNEL activities between the control and melatonin groups (Table 2).

**Table 2:** Immunohistochemical analysis

	Control	Melatonin	*p*-value
Caspase Stroma	1.61 ± 0.32	1.95 ± 0.33	0.48
Caspase Follicles	3.39 ± 0.75	2.37 ± 0.33	0.18
TUNEL Stroma	0.05 ± 0.01	0.21 ± 0.09	0.05
TUNEL Follicles	0.04 ± 0.01	0.07 ± 0.01	0.26

Immunohistochemical analysis comparing the use of melatonin added to the cryopreservation medium or not, in rat ovarian slide, freezing-thawing group (*n* = 3 in each group).

### Evaluation after transplantation

Ten animals in the melatonin and control groups were analyzed after autologous transplantation.

#### Estrous cycle evaluation

For all animals in both groups, there was a characterization of the estrus phase of the estrous cycle, demonstrating ovarian hormonal activity. The melatonin group presented faster resumption of the estrous cycle after transplantation (16.22 ± 0.50 days) compared with the control group (20.75 ± 1.89, *p* = 0.0017), indicating an enhancement in functional activity.

#### Morphology and morphometry

The melatonin group had an increase in mature follicle count compared to that in the control group, but no significant difference was observed in the immature follicles or in corpora lutea expression. With regard to immature follicles, we did not find any significant difference between the groups, even on the primordial follicular account.

**Fig. 1:**
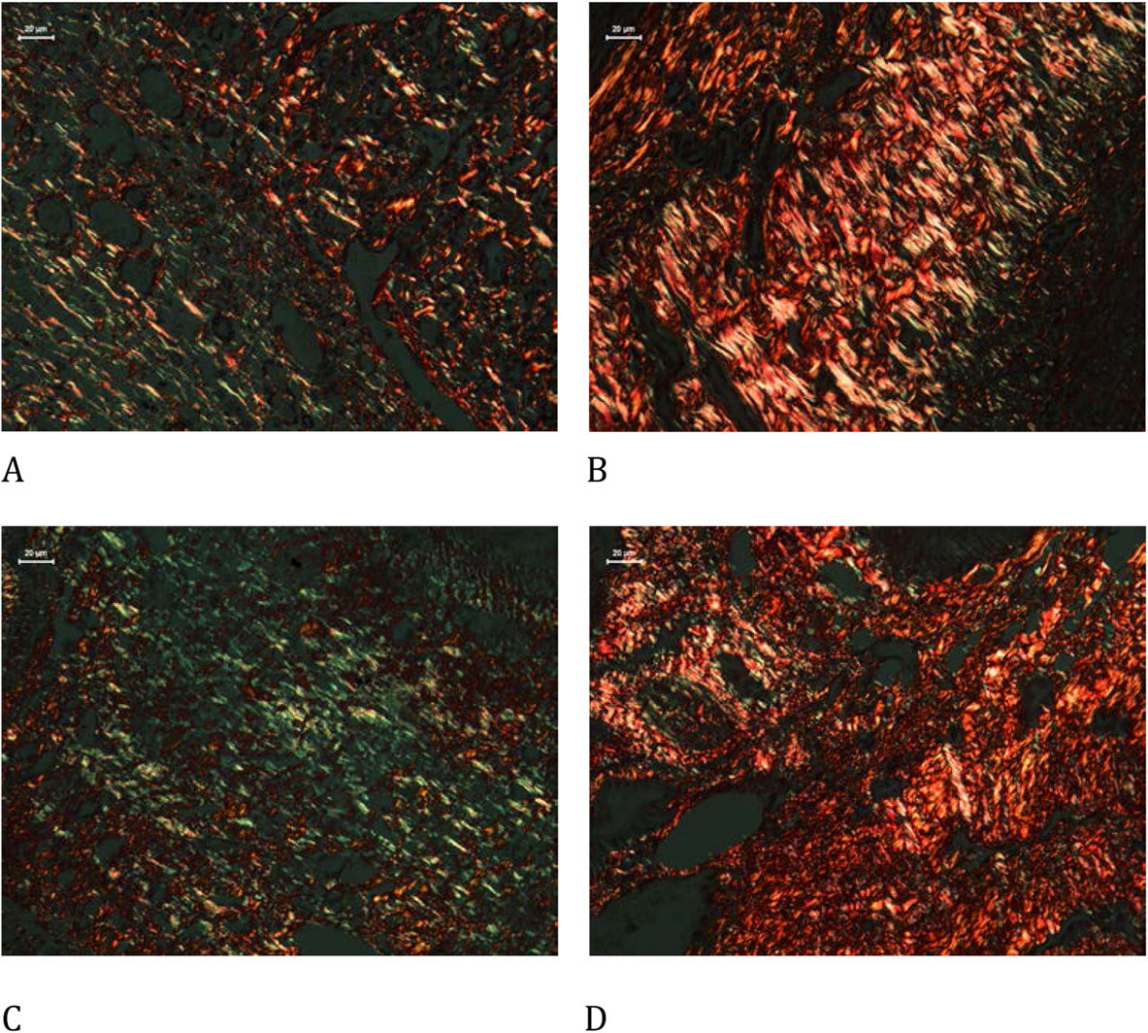
Picrosirius stain with polarized light analysis of types I and III collagen comparing the use of melatonin added to the cryopreservation medium or not, in ovarian rat autologous cryopreserved grafts 30 days after transplantation (*n* = 10 in each group); **a** control, type I collagen, 400×; **b** melatonin, type I collagen, 400×; **c** control, type III collagen, 400×; **d** melatonin, type III collagen, 400×

Blood vessel count did not differ significantly between the groups. The melatonin group showed enhancement and reduction in collagen type I and type III expression, respectively, compared to that in the control group (Table 3, Fig. 1).

**Table 3:** Histological analysis

	Control	Melatonin	*p*-value
Immature Follicles	7.20 ± 3.23	6.00 ± 2.55	0.66
Mature Follicles *	3.01 ± 0.91	8.75 ± 2.02	0.04
Corpora Lutea	7.86 ± 1.71	5.22 ± 1.05	0.34
Collagen type I*	5.75 ± 0.52	8.52 ± 1.04	0.03
Collagen type III*	3.53 ± 0.28	1.49 ± 0.15	< 0.001
Collagen type I and III ratio	1.62	5.71	
Blood vessels	20.13 ± 3.66	18.63 ± 3.65	0.99

Ovarian follicle density, collagen, and blood vessels (mean ± standard deviation) comparing the use of melatonin added to the cryopreservation medium or not, in rat ovarian slide, autologous cryopreserved grafts after the 30th day of transplantation (*n* = 10 in each group); *p < 0.05 *t*-test.

#### Immunohistochemistry

**Fig. 2:**
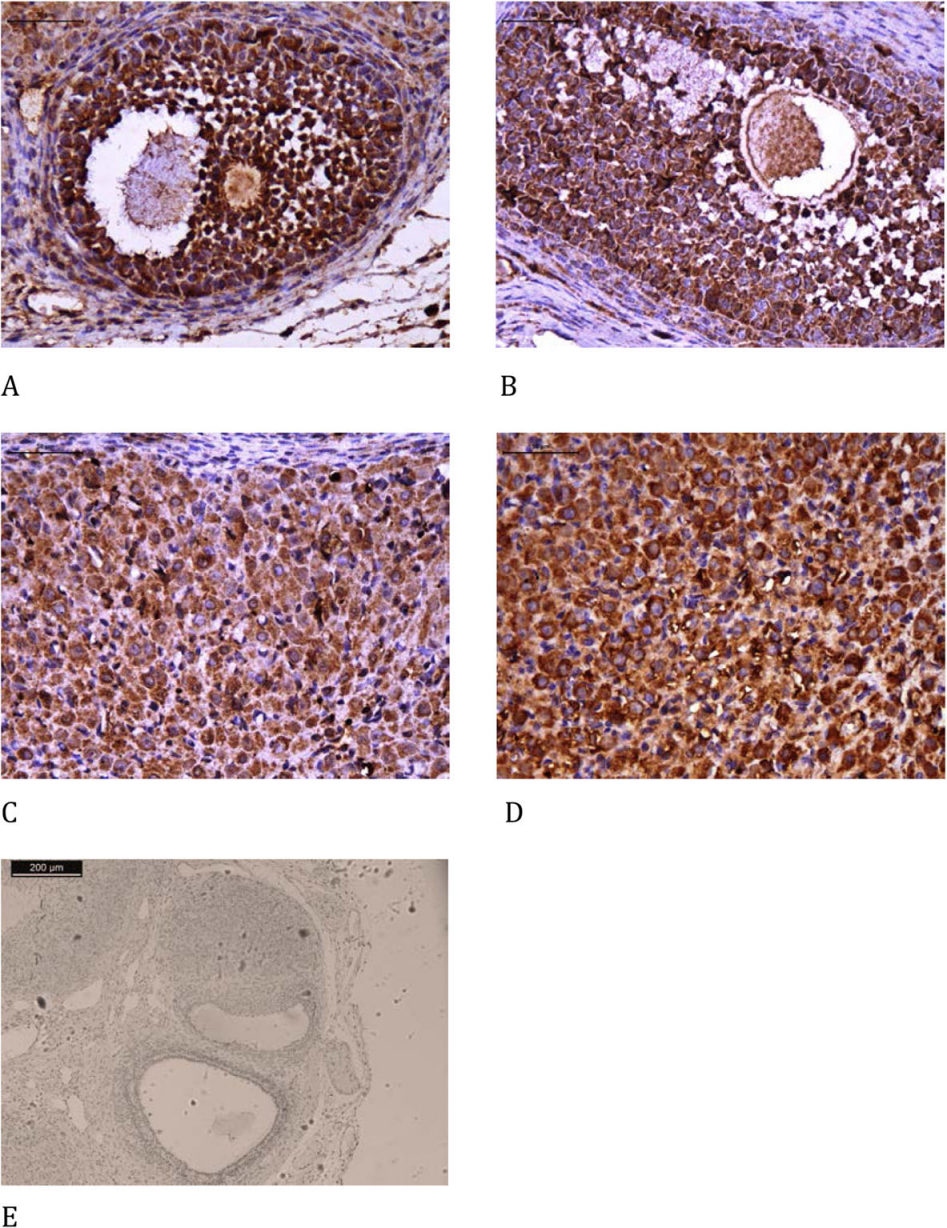
Immunohistochemical analysis of cleaved caspase 3 comparing the use of melatonin added to the cryopreservation medium or not, in ovarian rat autologous cryopreserved grafts 30 days after transplantation (*n* = 10 in each group); **a** control, follicle, 400×; **b** melatonin, follicle, 400×; **c** control, corpus luteum, 400×; **d** melatonin, corpus luteum, 400×; **e** negative control, 100×

**Fig. 3:**
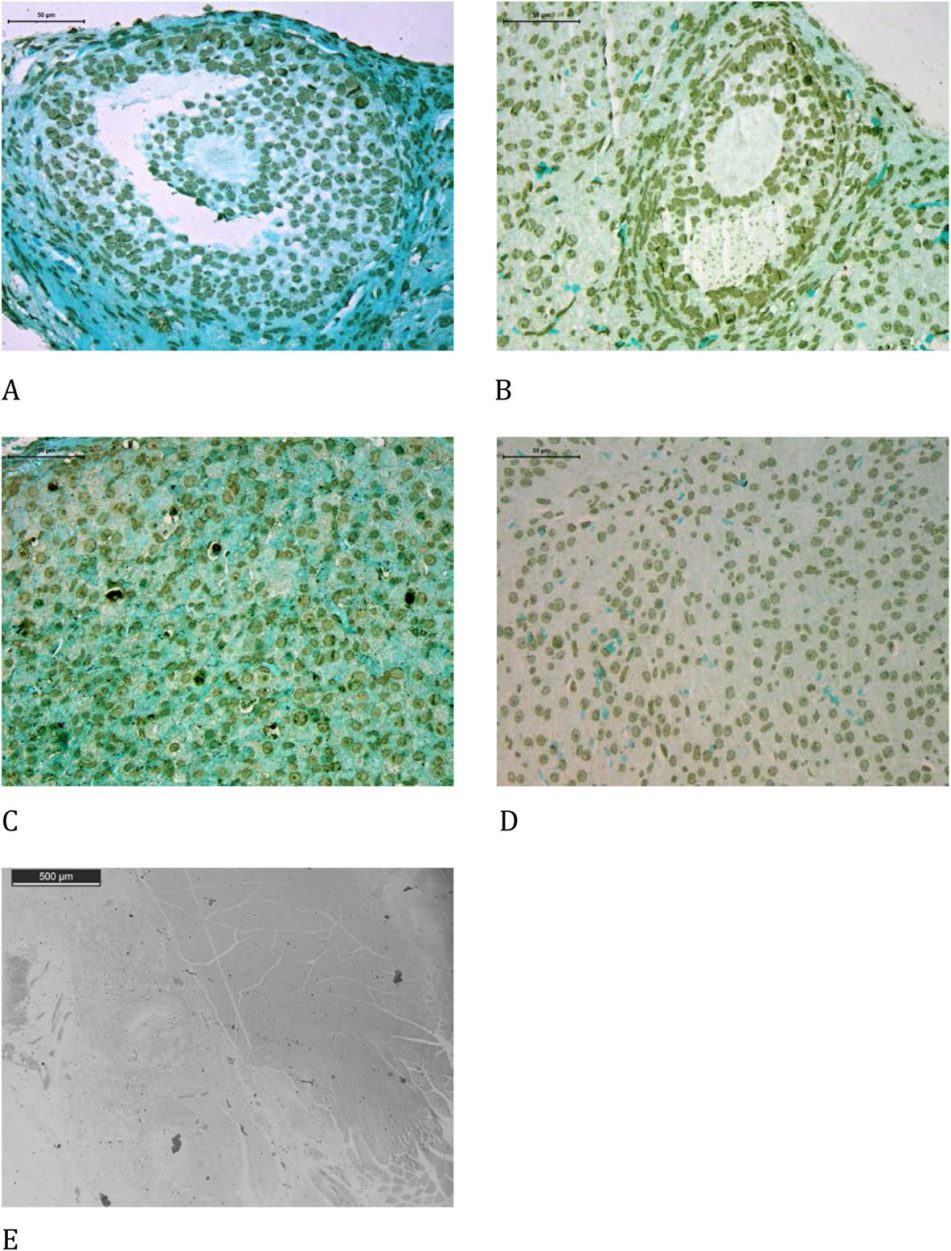
Immunohistochemical analysis of TUNEL comparing the use of melatonin added to the cryopreservation medium or not, in ovarian rat autologous cryopreserved grafts 30 days after transplantation (*n* = 10 in each group); **a** control, follicle, 400×; **b** melatonin, follicle, 400×; **c** control, corpus luteum, 400×; **d** melatonin, corpus luteum, 400×; **e** negative control, 100×

**Fig. 4:**
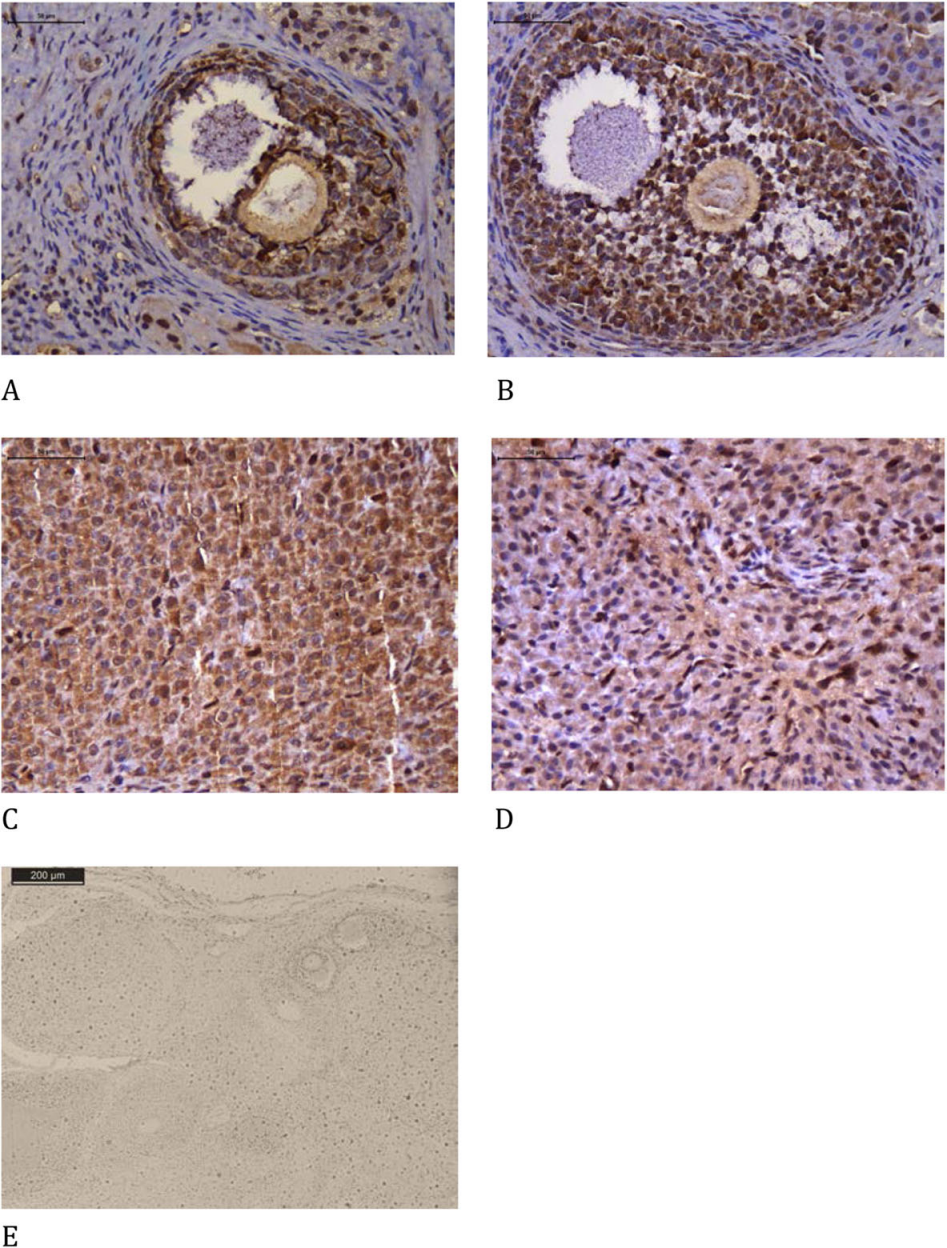
Immunohistochemical analysis of Ki-67 comparing the use of melatonin added to the cryopreservation medium or not, in ovarian rat autologous cryopreserved grafts 30 days after transplantation (*n* = 10 in each group); **a** control, follicle, 400×; **b** melatonin, follicle, 400×; **c** control, corpus luteum, 400×; **d** melatonin, corpus luteum, 400×; **e** negative control, 100×

**Fig. 5:**
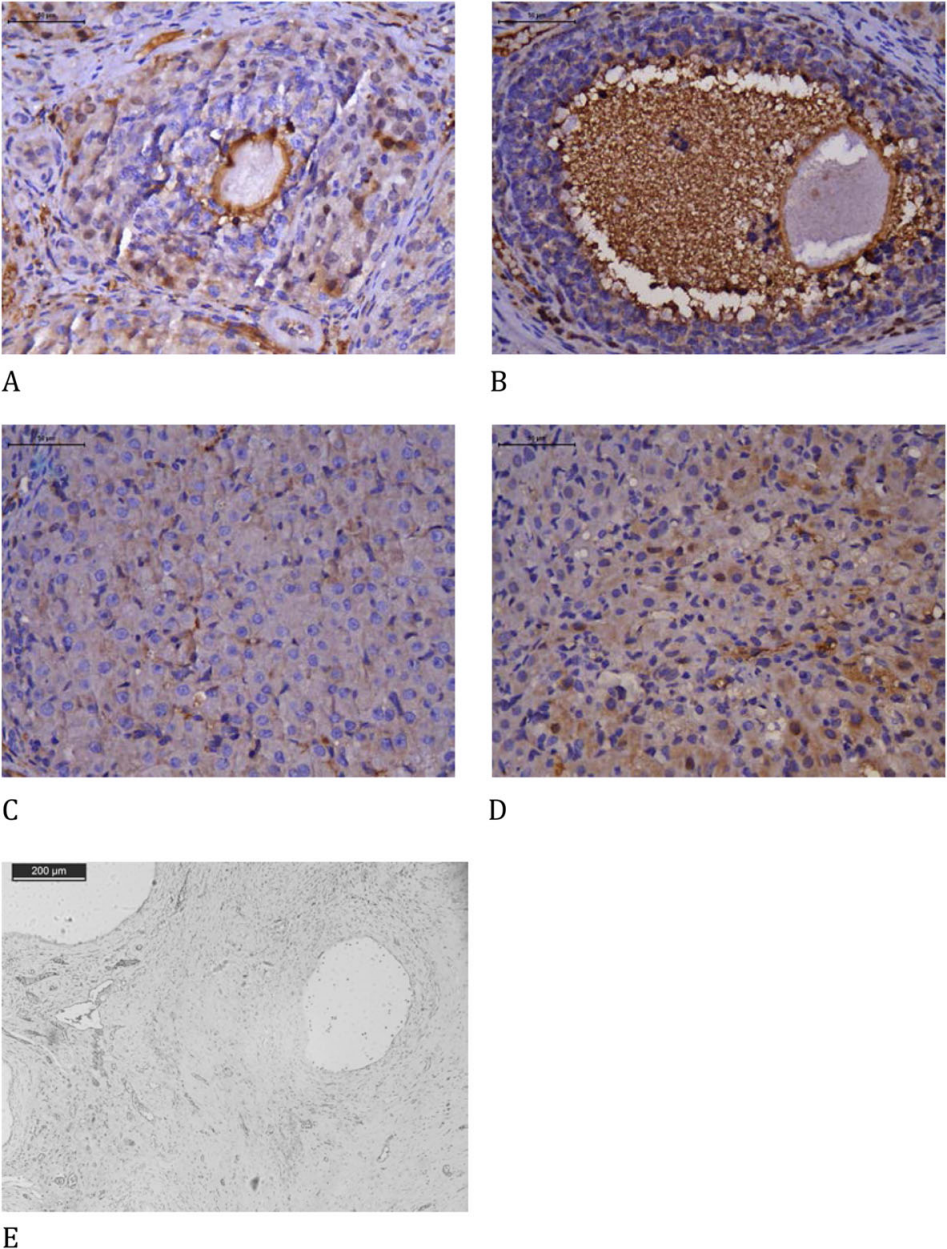
Immunohistochemical analysis of estrogen receptor comparing the use of melatonin added to the cryopreservation medium or not, in ovarian rat autologous cryopreserved grafts 30 days after transplantation (*n* = 10 in each group); **a** control, follicle, 400×; **b** melatonin, follicle, 400×; **c** control, corpus luteum, 400×; **d** melatonin, corpus luteum, 400×; **e** negative control, 100×

**Fig. 6:**
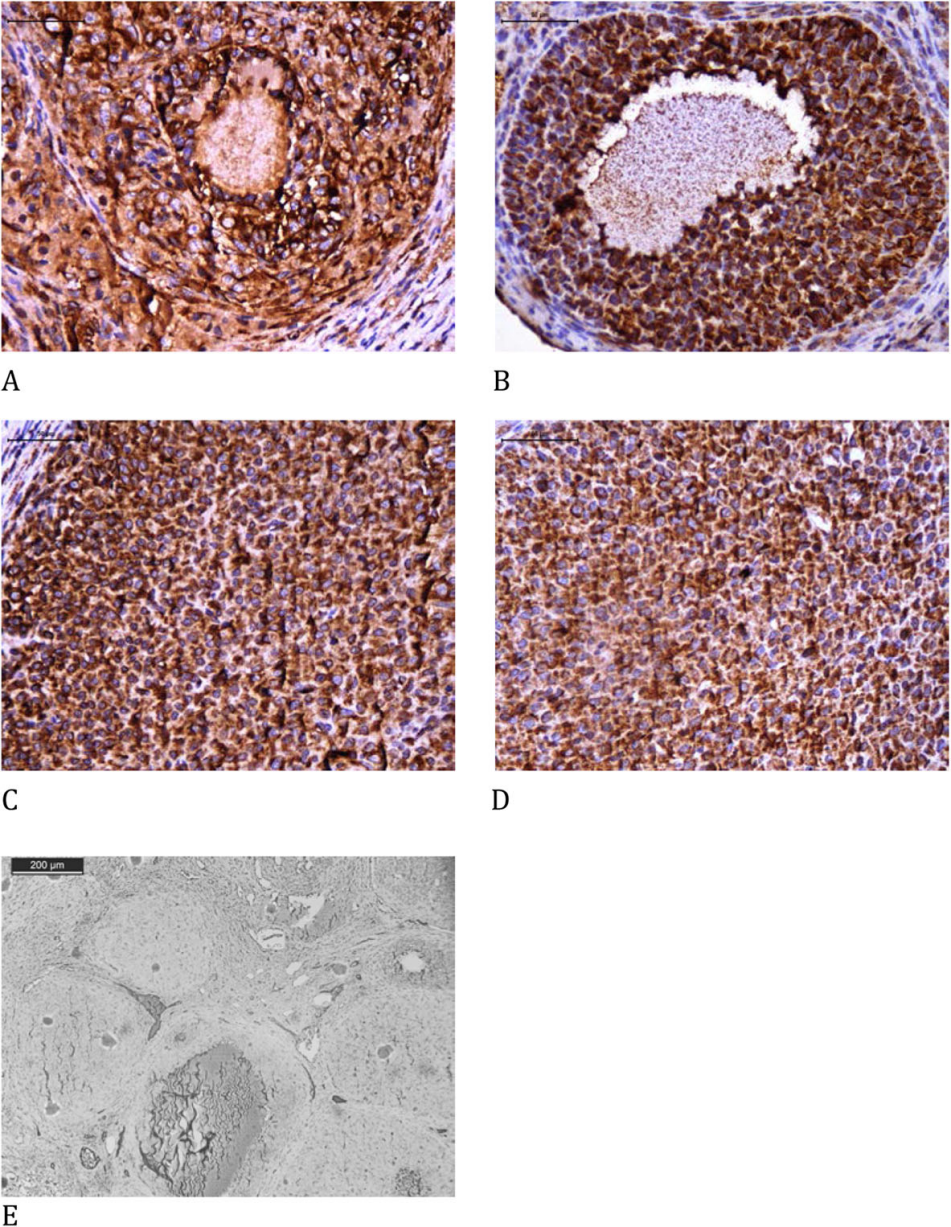
Immunohistochemical analysis of progesterone receptor comparing the use of melatonin added to the cryopreservation medium or not, in ovarian rat autologous cryopreserved grafts 30 days after transplantation (*n* = 10 in each group); **a** control, follicle, 400×; **b** melatonin, follicle, 400×; **c** control, corpus luteum, 400×; **d** melatonin, corpus luteum, 400×; **e** negative control, 100×

**Fig. 7:**
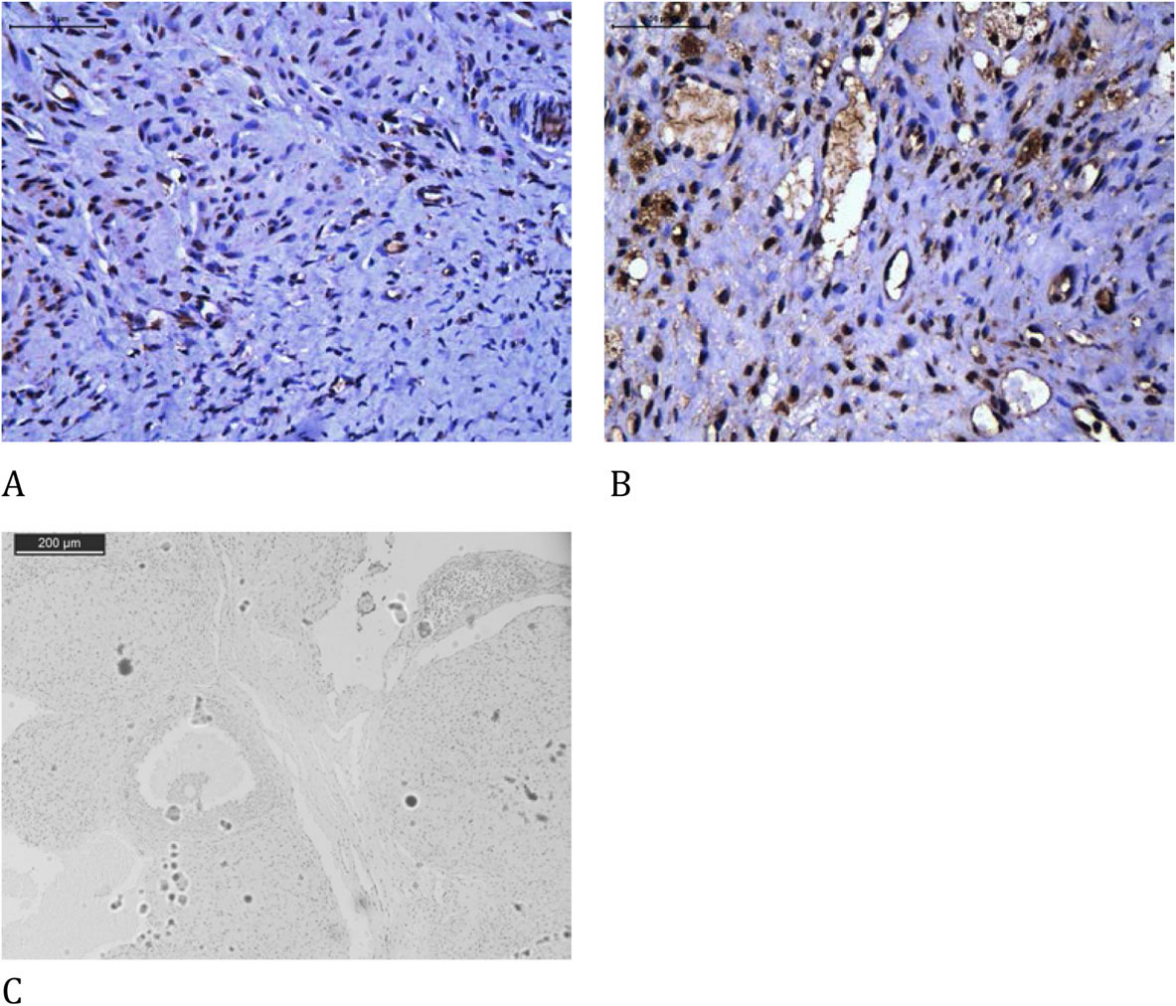
Immunohistochemical analysis of von Willebrand factor comparing the use of melatonin added to the cryopreservation medium or not, in ovarian rat autologous cryopreserved grafts 30 days after transplantation (*n* = 10 in each group); **a** control, 400×; **b** melatonin, 400×; **c** negative control, 100×

The melatonin group presented a significant increase in endothelial cells (vWF), cellular proliferation (Ki67), and estrogen receptors both on the follicles and corpora lutea, and apoptosis in the corpora lutea using the cleaved caspase-3 assay compared to that in the control group. There was a significant reduction in apoptosis using the TUNEL assay on follicles and corpora lutea. There were no significant differences in apoptosis using the cleaved caspase-3 assay on follicles and progesterone receptors on follicles or corpora lutea between both groups (Table 4, Figs. 2, 3, 4, 5, 6 and 7).

**Table 4:** Immunohistochemical analysis

	Control	Melatonin	***p***-value
**Cleaved caspase 3 F**	11.30 ± 1.87	13.70 ± 1.79	0.38
**Cleaved caspase 3 CL**	5.88 ± 0.82	24.50 ± 2.06	< 0.001*
**TUNEL F**	0.40 ± 0.14	0.04 ± 0.02	< 0.001*
**TUNEL CL**	1.58 ± 0.23	0.10 ± 0.03	< 0.001*
**Ki67 F**	1.46 ± 0.29	4.09 ± 0.55	0.002*
**Ki67 CL**	1.67 ± 0.33	5.27 ± 0.54	< 0.001*
**Estrogen receptor F**	2.41 ± 0.93	5.37 ± 0.73	0.02*
**Estrogen receptor CL**	2.25 ± 0.38	6.43 ± 0.85	< 0.001*
**Progesterone receptor F**	4.26 ± 0.53	4.65 ± 1.13	0.77
**Progesterone receptor CL**	16.62 ± 1.07	13.47 ± 1.72	0.14
**von Willebrand factor**	1.69 ± 0.28	3.19 ± 0.38	0.003*

Immunohistochemical analysis comparing the use of melatonin added to the cryopreservation medium or not, in rat ovarian slide, autologous cryopreserved grafts 30 days after transplantation (*n* = 10 in each group); * *p* < 0.05; *F* Follicle, *CL* Corpora lutea

## Discussion

Human ovary transplantation proceeds with cortex stripes and the present study performed whole ovary transplantation. Actually, previous experimental studies regarding melatonin on rat ovarian transplantation all performed whole ovaries techniques [25]. Rat ovaries are very small volume organs and cortex dissection would probably result in anatomical disruption. Besides, melatonin diffuses rapidly and easily through the different tissues [11].

Some studies have suggested the beneficial effects of melatonin on ischemic-reperfusion injury [11]. In the ovary, there are references to two specific receptors for melatonin (MT1 and MT2) [26] of the three identified so far, and they act via cyclic AMP reduction [27–29]. The same receptors have been identified in human ovarian follicles [27]. They act on ovarian function, modulating steroidogenesis, thereby notably increasing progesterone synthesis [29] and that of its receptor. They also modulate the formation of corpora lutea, indicating the optimization of ovulatory function [28]. Changes in serum hormone levels are related to ovulatory disorders in both rats and humans [27]. Melatonin-deprived rats have a persistent anovulatory cycle, premature vaginal opening, ovarian hypertrophy, and increased vaginal cell cornification. Moreover, melatonin replacement reverses these effects. The hormone is found in higher concentrations in the ovarian follicle as it develops, reaching maximum concentration, when compared to plasma in the preovulatory follicle [11, 13, 15]. The balance between free radicals and antioxidants in ovarian follicles appears to be fundamental for the proper functioning of oocytes and granulosa cells [11, 13, 15]. Moreover, melatonin reduction also decreases embryo implantation [27]. These are well-known effects of melatonin in the reproductive system. However, the effect of melatonin on free radicals and antioxidants to reduce the damage to the ovarian graft is a new direction in the use of this indolamine. Our study found that previous melatonin treatment on the graft was beneficial during the cryopreservation and transplantation of rat ovarian graft.

The first study on the effect of melatonin on rat ovarian autotransplantation reported the indirect benefits of intraperitoneal administration of the substance [30]. The authors described low levels of malondialdehyde as well as high superoxide dismutase and glutathione peroxidase levels with a low ovarian necrosis in melatonin-treated rats. Another study with animal xenotransplantation treated with melatonin via the oral route and vitamin E showed improvement in ovarian graft survival as evidenced by follicle count, the apoptosis index, and VEGF and PCNA expression [6]. None of these previous studies investigated the use of melatonin in the medium used for cryopreserving ovarian grafts.

Our results suggest that melatonin pretreatment in the cryopreservation medium also has beneficial effects on ovarian graft after transplantation: a) precocious resumption of the estrous cycle; b) a higher number of mature follicles; c) enhancement in tissue proliferation; and d) reduction in apoptosis.

The animals subjected to subsequent ovarian transplantation showed that the melatonin group demonstrated faster recovery of regular estrous cycles compared to the control. This finding suggests that melatonin could have enabled better conditions for ovulatory and hormonal activity recovery.

Histological analysis showed that the melatonin group presented more mature follicles than the control group, indicating that the indolamine promoted enhancement of graft function. In contrast, corpora lutea counting did not show any significant difference between groups. In rats, the estrous cycle can present corpora lutea formed by previous cycles, thus limiting quantitative comparison, which may explain this result. Blood vessel quantification did not differ between groups, and the melatonin group showed an enhancement in von Willebrand factor expression compared to that in the control. Previous studies, although not involving transplantation, have also reported such diverse effects of melatonin on blood vessel proliferation [18, 28]. Melatonin may have a modulatory effect on blood vessels in different biological scenarios [31].

Estrogen receptor expression was significantly higher in the melatonin group than in the control group. Indolamines can directly activate this hormone receptor activity or indirectly through the enhancement of follicular cell proliferation, which in turn synthesizes more estrogen. This hormone is the main representative activity of ovulatory follicles [32], thereby inferring that melatonin can enhance graft hormonal performance.

The present study is the first to assess progesterone receptors in transplanted rats and found no significant difference between the groups. An extended period of analysis may suggest that the application of melatonin to the cryopreservation medium could enhance progesterone receptor expression.

Melatonin can modulate the apoptosis process [33]. In the present study, the melatonin group showed an increase in cleaved caspase-3 on corpora lutea and no difference in follicles. For follicles, melatonin increased follicle development, which could be related to diminished cleaved caspase-3. For corpora lutea, the natural degradation process after ovulation might be related to the enhancement of cleaved caspase-3. This study is the first to analyze cleaved caspase-3 in ovarian rat autotransplantation treated with melatonin, and further studies might confirm how melatonin modulates cleaved caspase-3 activity in this scenario. DNA fragmentation analysis using the TUNEL assay is the final result of cell degeneration through the apoptosis cascade or not [34]. The present study revealed that TUNEL quantification was significantly reduced in the melatonin group, both in follicles and corpora lutea, compared with the control group. This finding relates to the most consolidated biochemical property of melatonin investigated in various tissues reported in the literature: the oxidative free radical scavenger, which could reduce DNA degradation, independently of the apoptotic pathway. Among experimental ovary transplantation studies, the first research to investigate the effect of melatonin also indicated a reduction in TUNEL [32].

This research revealed that the melatonin group showed enhancement in type I collagen and reduction in type III collagen compared to that in the control. Previous studies have related collagen expression to tissue response to hypoxia. Fibroblast activity in tissue cultures correlates with an increase in type I collagen [35] and a reduction in type III collagen [36] with better oxygenation. This result corroborates other reports of the present study, indicating that melatonin promoted better functional and follicular activity in the ovarian graft. The ovulation process is indeed related to the production and lysis of collagen in a dynamic process that ultimately produces ovarian follicle rupture and oocyte liberation [37].

## Conclusion

Melatonin may promote a better quality of ovarian grafts when used to evaluate histological and protein expression parameters. Future research can further assess the potential enhancement provided by melatonin in graft reproductive performance.

## Data Availability

The datasets used and/or analyzed during the current study are available from the corresponding author upon reasonable request.

## Abbreviations

PO: Postoperative
DMSO: Dimethyl sulfoxide
vWF: von Willebrand factor
TUNEL: Terminal deoxynucleotidyl transferase (TdT)-mediated dUTP nick-end labeling
MT: Receptors for melatonin
VEGF: Vascular endothelial growth factor
PCNA: Proliferating cell nuclear antigen
